# Extracting Diameter at Breast Height with a Handheld Mobile LiDAR System in an Outdoor Environment

**DOI:** 10.3390/s19143212

**Published:** 2019-07-21

**Authors:** Sanzhang Zhou, Feng Kang, Wenbin Li, Jiangming Kan, Yongjun Zheng, Guojian He

**Affiliations:** 1 Key Lab of State Forestry and Grassland Administration on Forestry Equipment and Automation, School of Technology, Beijing Forestry University, Beijing 100083, China; 2 College of Engineering, China Agricultural University, Beijing 100083, China; 3 Dalian Hangjia Robotics Co, Ltd., Dalian 116000, China

**Keywords:** mobile laser scanning, 3D point cloud map, diameter at breast height

## Abstract

Mobile laser scanning (MLS) is widely used in the mapping of forest environments. It has become important for extracting the parameters of forest trees using the generated environmental map. In this study, a three-dimensional point cloud map of a forest area was generated by using the Velodyne VLP-16 LiDAR system, so as to extract the diameter at breast height (DBH) of individual trees. The Velodyne VLP-16 LiDAR system and inertial measurement units (IMU) were used to construct a mobile measurement platform for generating 3D point cloud maps for forest areas. The 3D point cloud map in the forest area was processed offline, and the ground point cloud was removed by the random sample consensus (RANSAC) algorithm. The trees in the experimental area were segmented by the European clustering algorithm, and the DBH component of the tree point cloud was extracted and projected onto a 2D plane, fitting the DBH of the trees using the RANSAC algorithm in the plane. A three-dimensional point cloud map of 71 trees was generated in the experimental area, and estimated the DBH. The mean and variance of the absolute error were 0.43 cm and 0.50, respectively. The relative error of the whole was 2.27%, the corresponding variance was 15.09, and the root mean square error (RMSE) was 0.70 cm. The experimental results were good and met the requirements of forestry mapping, and the application value and significance were presented.

## 1. Introduction

With the development of light detection and ranging (LiDAR) technology, researchers in various fields began to apply it for modelling analysis. In the field of aerospace assembly, horizontal docking assembly is a fundamental process. Zhang et al. [1] segmented the point cloud of the cylindrical parts obtained by a 3D laser scanner into a series of parallel planar profiles, and ellipse fitting was employed in order to estimate each center of the section profile. The pose of the whole part can be obtained through a spatial straight line fitting with these profile centers. A robust method improved from M-estimation and RANSAC was developed, avoiding the influence of the interference features on the surface of the parts in the practical assembly process. Pawel Burdziakowski [2] merged laser scanning data and images together using the iterative closest point (ICP), and the structure-from-motion (SfM) algorithms, respectively, for modelling ship hulls in unfavorable conditions, and an accuracy of 1 cm was achieved. Extracting tree characteristics from the three-dimensional maps of trees in forest areas based on LiDAR equipment is of great significance for forest inventories [3,4]. Accurate 3D mapping relies on estimates of the position and pose during platform movement [5]. In mobile laser scanning (MLS), inertial measurement units (IMU) is used in conjunction with Global Navigation Satellite Systems (GNSS) for the position and pose estimation [6,7].

Erzhuo Che et al. [8] discussed in sufficient depth the processing methods of MLS point clouds in different scenes. The benchmark datasets for the classification, recognition, and metrics used to assess the quality of the processing were summarized. The challenges of dealing with point clouds, and the future development trend were illustrated. Liang et al. [9] built an MLS system for forest inventories. The system was composed of a high-performance laser scanner, a navigation unit, and a six-wheeled all-terrain vehicle. In the experiment, approximately 0.4 ha of forest area was mapped utilizing the MLS system. The stem mapping accuracy was 87.5%, and the root mean square errors (RMSEs) of the estimates of the diameter at breast height and the location were 2.36 cm and 0.28 m, respectively. These results indicated that the MLS system has the potential to accurately map large forest plots, and further research on mapping accuracy and cost–benefit analyses is needed. Marek Pierzchała [10] employed Velodyne VLP-16 LiDAR, a stereo camera, IMU, and Global Positioning System (GPS) to install the device on a mobile platform to form the MLS system. The experimental area was approximately 900 m^2^. The authors segmented and clustered the 3D point cloud map, and fitted the tree point cloud in order to obtain an average error of 2 cm (7–12%) and an RMSE value of 2.38 cm (9%); the positioning error was 0.0476 m. Mónica Herrero-Huerta [11] used a MLS system consisting of Riegl VQ250 laser scanners, all-terrain vehicles, and a navigation system in order to generate a three-dimensional point cloud map of 58 trees along a road, and to measure the relevant structural parameters, with an RMSE of 4.9 cm for the diameter at breast height (DBH). Juraj Čerňava et al. [12] exploited GNSS time-based clustering in order to process the MLS data of forests scanned from different views with two mobile laser scanners under a heavy canopy. An OPALS ICP module was used to align the overlapping clusters, and the root-mean-square error of the estimated DBH was 3.06 cm. Liu et al. [13] extracted and estimated the DBH of individual trees in regions with a complex terrain based on the point cloud data obtained by terrestrial laser scanning (TLS). The extraction of the DBH and height of trees were realized by Octree segmentation, connected component labeling, and random Hough transform. The results showed that the topography, undergrowth shrubs, and forest density influence the scanning range of the plots and the accuracy of feature extraction. The average RMSE of the DBH and tree height were 1.28 cm and 0.95 m, respectively.

Some researchers have also explored the use of 2D lasers to generate 3D point cloud maps. A two-dimensional sick laser and GNSS was used by André F. Colaço [14] in order to generate a three-dimensional point cloud map of a citrus orchard. The two-dimensional laser was perpendicular to the measured canopy. Combined with GNSS data, a three-dimensional point cloud was generated. The image was filtered by a distance threshold. The K-means algorithm was used in the clustering operation, and the volume and height of the canopy were calculated by a α-shape algorithm. The proposed system was useful for site-specific management in orange groves. James P. [15] utilized a ground mobile platform with a two-dimensional LiDAR and a color camera in order to generate a two-dimensional map of an almond orchard. GPS/INS position calibration was used to generate a three-dimensional point cloud, which was segmented using the Markov random field probabilistic ground model. The system was able to efficiently map flower and fruit distributions, and estimate and predict the yields for individual trees. Escolà, A [16] used a 2D laser to generate a 3D point cloud map of an olive orchard, and different algorithms were developed in order to estimate the canopy volume, which was comparable with traditional methods. The results proved that these approaches were equivalent with coefficients of correlation ranging from r = 0.56 to r = 0.82, depending on the algorithms used.

In recent years, the improvement of unmanned aerial vehicle (UAV) technology has led to this technology playing an increasingly important role in 3D forest mapping. Peter Krzystek [17] combined LIDAR data with aerial images obtained using cameras with multispectral sensors, and used machine learning and computer vision methods to detect and classify forest objects. Dr. Krzystek and his team found that they could classify coniferous and deciduous trees with excellent accuracy, and his team was able to classify four tree species with fairly good accuracy, by combining a large set of features generated from aerial imagery and multispectral LiDAR. Li et al. [18] exploited UAVs with GNSS and IMU-aided SfM for trajectory estimation, and used the two-step adaptive extraction method to segment and estimate the tree height and canopy diameter. The results illustrated the high potential of low-cost unmanned laser scanning (ULS) in 3D forest mapping.

Segmentation and detection are key techniques for extracting object features from 3D maps. Ma et al. [19] summarized some state-of-the-art commercial MLS systems (such as, MX-9, VMX-450, VMX-2HA, Lynx SG, StreetMapperIV, IP-S3 Compact+, and ROAD SCANNER-C), and analyzed detection and extraction methods for on-road objects (e.g., road surface, road markings, driving lines, and road crack) and off-road objects (e.g., pole-like objects and power lines). The challenges and future trends of the MLS technique were discussed. Gargoum et al. [20] proposed a voxel-based method for detecting light poles on rural highways. The data was voxelized so as to classify them into ground and non-ground points. Connected components labeling was used to perform 3D clustering of the data voxels, and a density-based method was applied to combine the connected components of the same object; finally, light poles were extracted using the geometric properties of the clusters. Yu et al. [21] presented an algorithm for detecting road scene objects. The local features were described by a contextual visual vocabulary generated by integrating the spatial contextual information of feature regions. Objects of interest were detected based on local features; the proposed algorithm was tested in two data sets, and achieved an average recall, precision, quality, and F-score of 0.949, 0.970, 0.922, and 0.959, respectively. Shi et al. [22] offered a method for extracting and classifying pole-like objects (PLOs) from unstructured MLS point cloud data. Raw point cloud downsampling was performed, and the outliers and ground points were removed. The PLOs were extracted by spatial independence analysis and cylindrical or linear feature detection, and were classified by 3D shape matching. The correctness of testing on the two datasets was 97.4% and 97.1%, respectively. The method based on deep learning has also been applied in the field of point cloud segmentation and classification. Griffiths et al. [23] reviewed the current state-of-the-art deep learning architectures for processing unstructured Euclidean data, including red, green, blue and depth map (RGB-D), multi-view, volumetric, and fully end-to-end architecture designs. A detailed discussion about the future of deep learning for 3D sensed data was given. Zou et al. [24] proposed a voxel-based deep learning method to classify tree species in 3D maps. Individual trees were extracted by point cloud density, and voxel-based rasterization was utilized in order to obtain features. Two data sets were tested with a deep learning model, and achieved an average classification accuracy of 93.1% and 95.6%, respectively. Guan et al. [25] employed the deep learning-based approach to handle mobile LiDAR data. The ground points were removed by voxel-based upward-growing filtering, and individual trees were segmented by Euclidean distance clustering and voxel-based normalized cut segmentation. Waveform representation and deep Boltzmann machines were applied in order to classify trees. An overall accuracy of 86.1%, and a kappa coefficient of 0.8 were achieved when classifying the urban tree species.

Simultaneous localization and mapping (SLAM) is the major technology for generating 3D maps, and is a process that can simultaneously generate a map of unfamiliar environmental conditions and locate a locating the mobile platform [26,27]. Some researchers applied SLAM technology in the forestry field in order to carry out forestry inventories [5,10,28,29,30]. Marek Pierzchała [10] utilized the graph-SLAM algorithm to generate forest maps, and extracted tree diameters and locations. Tsubouchi T [30] selected several measurement points to collect point cloud information, and used SLAM technology and the frame-to-frame matching algorithm to create a three-dimensional map of the forest area by ICP so as to extract the structural characteristics of forest trees. Antero Kukko [28] combined IMU and GNSS in the MLS system, using a graph optimization method to calibrate tracks and generate 3D maps of the forest areas. The results showed that the author could improve the internal conformity of the data significantly from 0.7 cm to 1 cm, based on the tree stem feature location data. When the optimization result was compared to a reference at the plot level, it was shown that the author had achieved a mean error of 0.06 m for the absolute tree stem locations.

Today, the most famous open-source SLAM algorithms are Google’s Cartographer 3D [31], Berkeley’s open source code Berkeley Localization and Mapping (BLAM) [32], and the LiDAR odometry and mapping (LOAM) algorithm [33,34]. After comprehensively comparing several algorithms, a LOAM algorithm combined with Velodyne VLP-16 LiDAR and IMU was used to design a handheld 3D scanning device in order to generate a 3D environment map in real time.

A designed handheld 3D scanning device was exploited to generate a 3D map of the forest environment. The experimental area had a perimeter of 130 m and contained 71 trees. The RANSAC algorithm was used to remove the ground point cloud in the experimental area. The trees in the experimental area were segmented by the European clustering method [35]. The PassThrough filtering method [36] was used to extract the interval, and it was projected onto a two-dimensional plane. It was supposed that the cross section of the tree trunk could be described as a circle and the diameter of the circle was considered as the DBH; the RANSAC algorithm was used to fit the circle, and the DBH value was obtained.

## 2. Materials and Methods

### 2.1. Hardware and Software Setup

The designed handheld 3D scanning device consisted of a laser scanner (VLP-16 LiDAR, Velodyne Lidar Co., San Jose, CA, USA), an IMU (Mti-30-2A8G4, Xsens Co., Enschede, Holland), and a micro-computer (BRIX GB-BRi7H-8550, GIGABYTE Co., Zhejiang, China). The VLP-16 has a 360° horizontal and 30° vertical field of view. The laser beams have a divergence of 3 mrad. Full 360° scans of 300,000 points were acquired with a frequency of 2 Hz. The maximum range of the VLP-16 is up until 100 m, depending on the reflectivity of the target surface [9]. According to the vendor manual, the scanner operates with a typical accuracy of ±3 cm. Typical accuracy refers to the ambient wall test performance across most channels, and may vary based on factors, including, but not limited to, range, temperature, and target reflectivity. The main parameters of the microcomputer are as follows: Intel i7 8550U quad-core eight-thread Central Processing Unit (CPU), 8G Double-Data-Rate Fourth Generation Synchronous Dynamic Random Access Memory (DDR4 SDRAM), and a 256G Solid State Drive (SSD) hard disk. The microcomputer collects the original data and runs the core 3D laser SLAM algorithm, which finally generates the point cloud and the motion trajectory in the global coordinate system. The origin of the global coordinate system is the starting position of the LiDAR motion. IMU is used to assist the measuring equipment so as to obtain the motion posture and displacement information in a short period, thereby providing a reliable initial value for the 3D SLAM algorithm [37].

**Figure 1:**
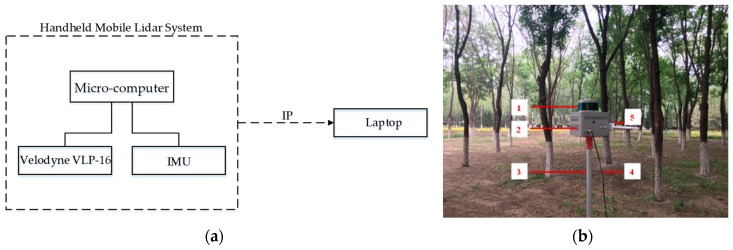
The mobile measurement system workflow chart and components. (**a**) System workflow chart; (**b**) system component. (1) Velodyne VLP-16 light detection and ranging (LiDAR); (2) encapsulated shell; (3) hand-held pole; (4) power cable; (5) wireless module.

The microcomputer runs a driver for a sensor, such as a laser radar sensor, an IMU, a SLAM program, and an interactive communication program with a notebook client for receiving client commands over the network, or transmitting the collection point cloud to the client. All of the programs are written in C++, run on the Ubuntu 16.04 operating system, and rely on the Robot Operating System (ROS). The mobile measurement system workflow chart and components are shown in Figure 1.

The laptop runs the client software 3D_Viewer, sends control commands to the designed handheld 3D scanning device via a wireless or wired network, and generates a 3D display based on the global point cloud returned by the designed handheld 3D scanning device. The client software is written in C++ based on the Qt interface programming framework. The 3D display function is based on OpenGL development, and the entire program runs on the Windows system by default.

### 2.2. Experimental Area

**Figure 2:**
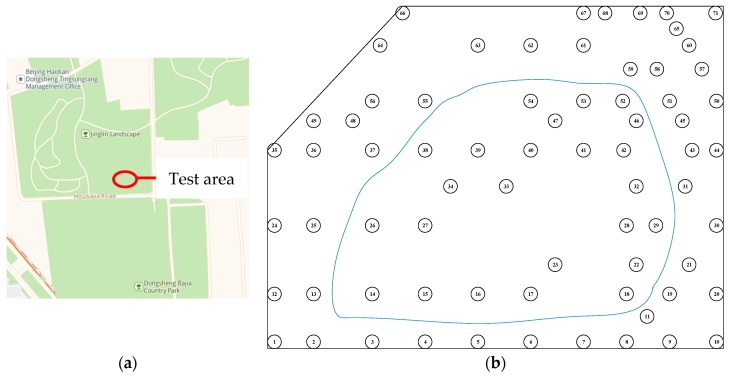

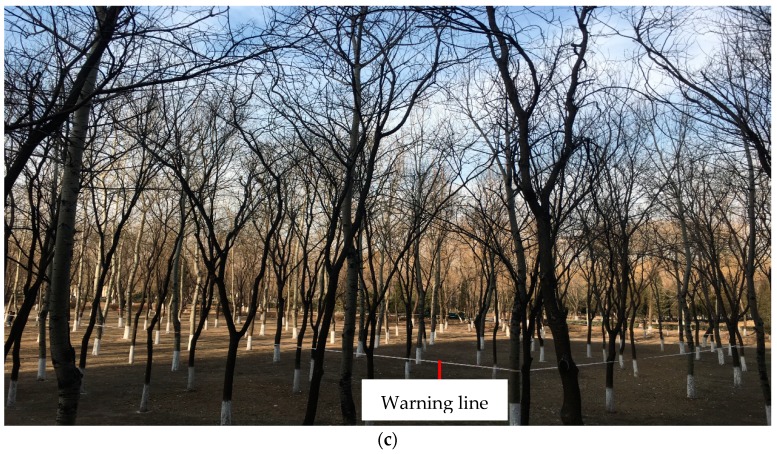
Experimental area. (**a**) Geographical location; (**b**) schematic representation; (**c**) experimental setup.

The experimental site is located in Bajia Park, Haidian District, Beijing. The experimental area is surrounded by a warning line. The perimeter is approximately 130 m. The warning line is approximately 2 m from the ground. There are 71 trees in the experimental area, including 40 eucalyptus trees and 31 poplar trees. The average DBH of the trees is 20.93 cm, the minimum DBH is 11.75 cm, and the maximum is 32.78 cm. The geographical location, schematic diagram, and experimental setup of the experimental area are shown in Figure 2. In the diagram, the blue solid line is the motion trajectory.

### 2.3. Extraction of DBH from the SLAM Map

**Figure 3:**
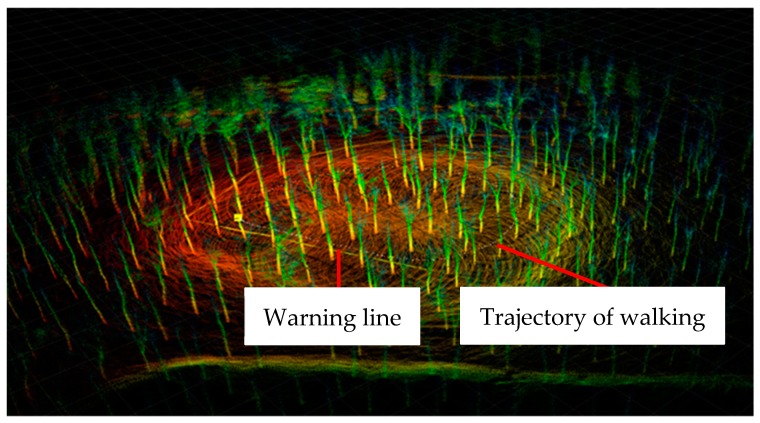
3D point cloud map of the forest area.

In the experimental area, the experimenter walks in a circle using the designed handheld 3D scanning device, and the LiDAR device senses the surrounding environment, and generates a three-dimensional point cloud map of the forest area according to the SLAM algorithm. The 3D point cloud map of the forest area is shown in Figure 3. The white dotted line indicates the trajectory of walking, and the solid line is the warning line. According to the 3D point cloud map, part of the point cloud map was outside of the experimental area. In order to remove the parts outside of the experimental area surrounded by the warning line, a polygon tool was used in an open source software named CloudCompare, in order to crop the 3D point cloud map of the experimental area.

In the three-dimensional point cloud map of the polygon, the ground and trees are not separated, which will have a certain impact on the clustering and segmentation of the trees. This study uses the Point Cloud Library (PCL) [38] to write programs that segment the ground and trees.

**Figure 4:**
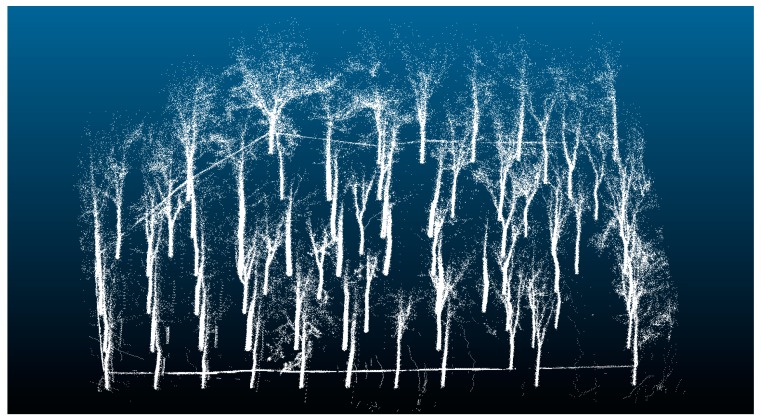
3D point cloud map without the ground area.

The RANSAC algorithm is widely used in plane fitting and the segmentation of point clouds [39,40,41]. The study uses the RANSAC algorithm to fit the plane model, and the point cloud belonging to the ground is classified as a plane model. After plane fitting, segmentation is performed. The processed point cloud is shown in Figure 4.

After removing the ground point cloud, it was found that the trees in the experimental area were not individually separated, and instance segmentation of the trees was required.

**Figure 5:**
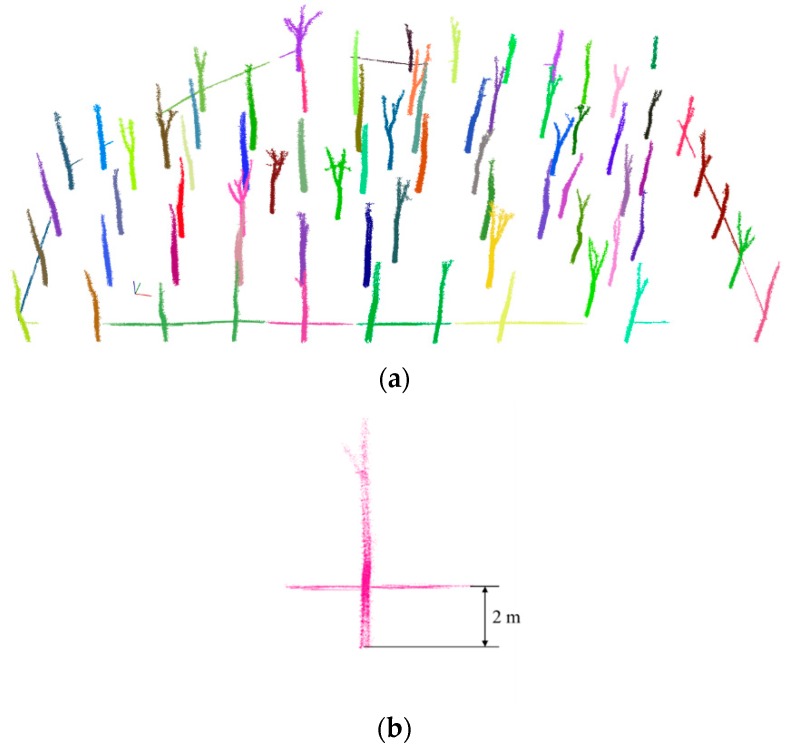
Clustering and individual analysis. (**a**) Clustering results for the experimental areas; (**b**) tree number 5.

The common conventional point cloud clustering segmentation algorithms are region growing segmentation [42], min-cut based segmentation [43], difference of normal-based segmentation [44], super voxel-based segmentation [45], progressive morphological filter segmentation [46], and Euclidean cluster extraction algorithms [35,47]. Considering the spatial distribution characteristics of the trees in the forest, and the clustering effect, parameter adjustment, and time-consuming of nature other algorithms, this study finally used the Euclidean cluster extraction algorithm to segment trees in the forest areas. The segmentation result for the experimental area is shown in Figure 5a, and each color in the figure represents a category. Although some trees and warning lines fall into one category, such as tree number 5, as shown in Figure 5b, this does not affect the extraction of the DBH point cloud, because the distance between the warning line and the ground is approximately 2 m, and the warning lines will not interfere with the point cloud in the DBH section.

**Figure 6:**
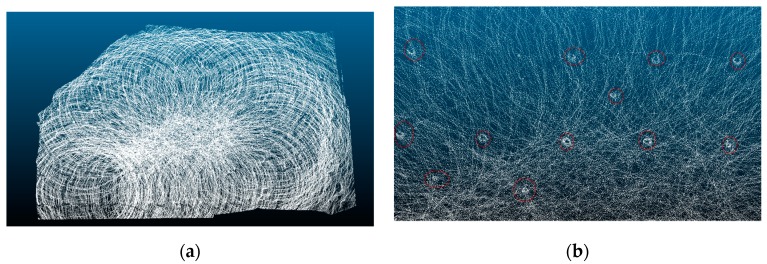
Overall and partial map of the ground point cloud. (**a**) Overall map of the ground point cloud; (**b**) partial enlargement of the ground point cloud, the removed ground point cloud contains the trunk portion, and the red circle is marked.

Usually, a measurement should be taken at a distance of 1.3 m from the ground for measuring the DBH of trees. Because of the undulation of the ground in the experimental area, and the unavoidable shaking in the point data collection process using the designed handheld 3D scanning device, the point cloud of the removed ground contained the lowest portion of some trees, as shown in Figure 6a,b. In order to guarantee the accuracy of the DBH estimation, three segment intervals of 1.0–1.1, 1.1–1.2, and 1.2–1.4 m of the point cloud data in Figure 6a were chosen by the PassThrough filtering method in order to estimate the DBH, and the interval with the smallest error was selected as the final estimation result. The cross section of the tree trunks was described as a circle and the diameter of the circle was considered as the DBH. In an interval segment, all of the tree point clouds were projected onto the lowest two-dimensional plane. The 1.0–1.1, 1–1.2, and 1.2–1.4 m intervals corresponded to planes of 1.0, 1.1, and 1.2 m from the ground.

**Figure 7:**
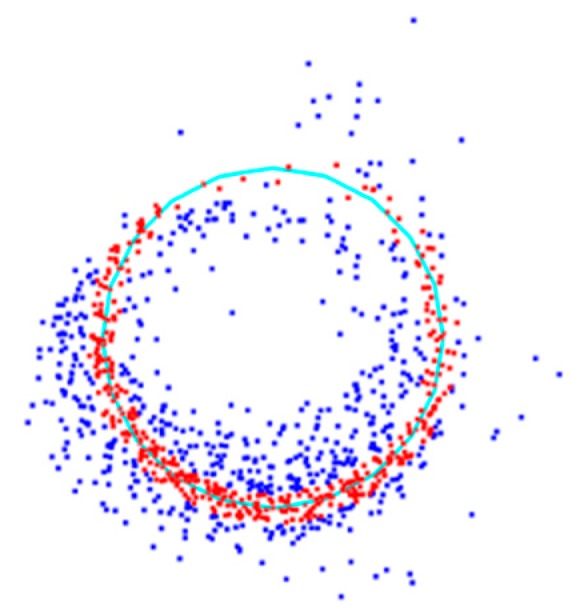
A 1.2–1.4 m interval point cloud of tree number 14 was projected onto a two-dimensional plane. Red points are considered to be inliers, while blue points are outliers, the proportion is 57.2%, and the fitted circle is cyan.

The point cloud on a 2D plane was fitted into a circle. So far, there are several methods (e.g., Shift-Msplit estimation [48], Msplit(q)–estimation [49], the alpha-shape [50], and RANSAC algorithm [51]) to fit a circle based on point cloud data in a two-dimensional plane. Zienkiewicz, M. H. proposed the Shift-Msplit estimation method based on the additional random variables generated by the outliers. Seven point cloud models in the form of circular equations on a two-dimensional plane were established. Under the premise of knowing the number of models, the Msplit(q)-estimation method was used to cluster and estimate the center coordinates and the radius of the point cloud by Artur Janowski. The alpha-shape algorithm was also utilized to fit a circle by the same author. The RANSAC algorithm is robust to the outlier, and only the setDistanceThreshold and setRadiusLimits parameter values need to be adjusted. In this research, the RANSAC algorithm was used to fit the circle on a 2D plane. Suppose the fitted model is a circle, setDistanceThreshold represents the distance threshold from the points to the fitted circle. The points whose distance to smaller than the threshold are considered to be inliers or outliers, as shown in Figure 7. SetRadiusLimits represents the radius range of the fitted circle.

In order to explore the influence of the number of inliers on the estimation DBH, credibility (C) was defined as the ratio of the number of inliers to the total number of point clouds. Through an analysis of the estimation of the DBH and real values for the trees in the experimental area, the relationship between credibility and error was explored. As shown in Figure 7, credibility is visualized in the fitted circle.

## 3. Results

A three-dimensional map of the forest was generated with the designed handheld 3D scanning device, and the software was used to segment the experimental area in the three-dimensional map. The corresponding program was written to successfully segment the tree; three different intervals were extracted and projected separately onto the 2D plane in order to fit the radius of a circle.

**Figure 8:**
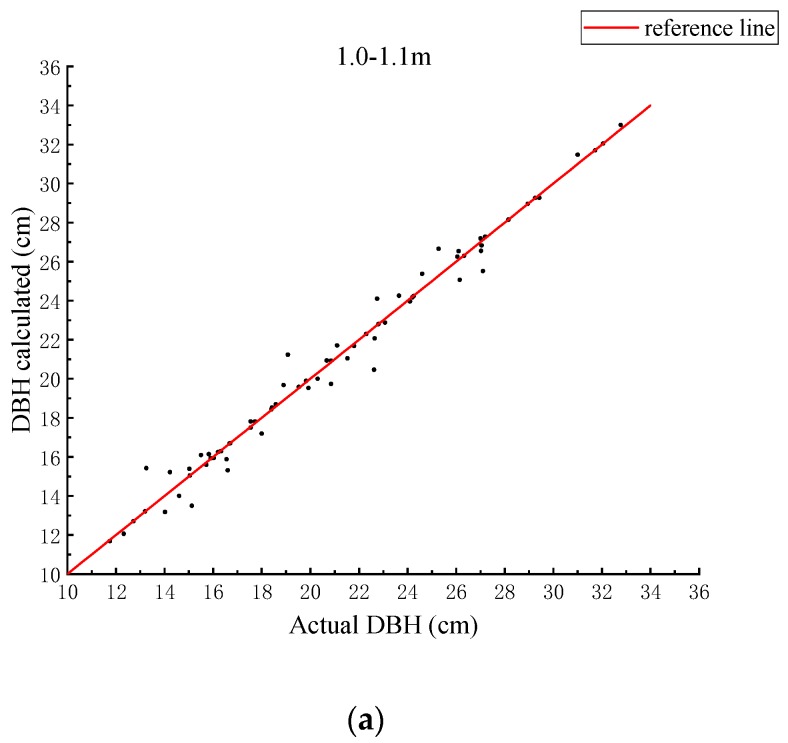

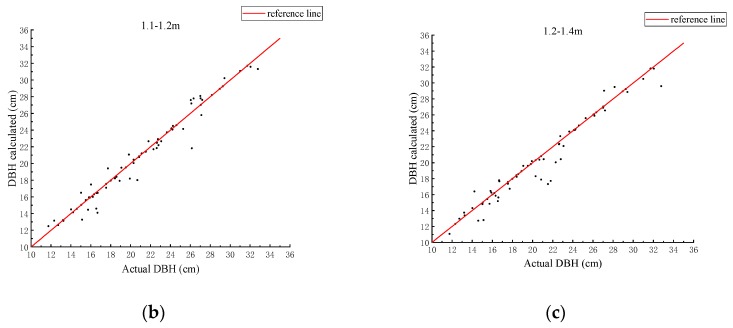
Fitting results and true values for different intervals. (**a**) Interval of 1.0–1.1 m; (**b**) interval of 1.1–1.2 m; (**c**) interval of 1.2–1.4 m.

Considering the error of the data processing, three different intervals of point clouds were selected as the area where the DBH was located, and were projected onto the two-dimensional plane to fit the radius of a circle. The fitting result and the true value of each segment are plotted in Figure 8. The red reference line in Figure 8 is the quadrant bisector. In Figure 8a, the points in the figure are evenly distributed on both sides of the reference line, and the distance to the reference line is short. In Figure 8b, the distance of the point from the reference line is larger than in Figure 8a. In Figure 8c, the distance of the point in the figure from the reference line is greater than that in Figure 8a,b.

**Figure 9:**
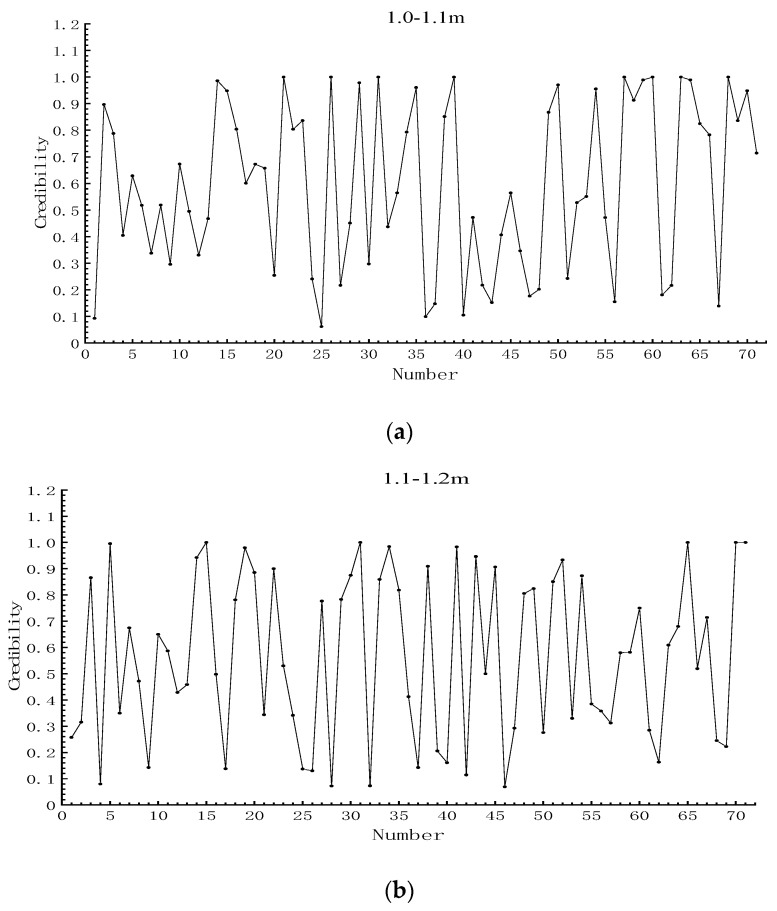

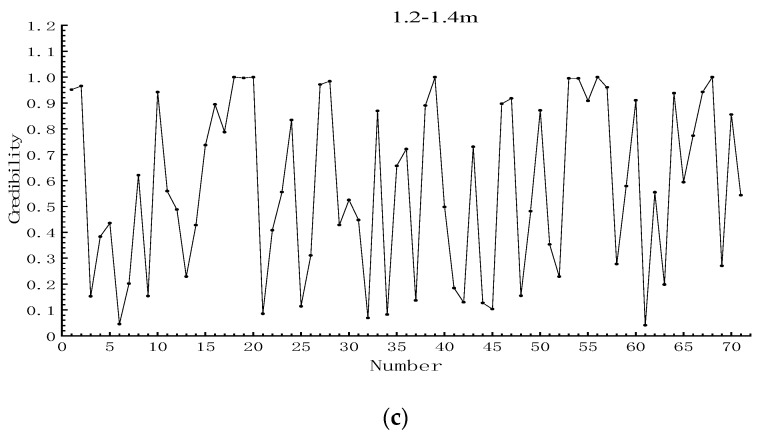
Distribution of credibility in different intervals. (**a**) Distribution of credibility in the 1.0–1.1 m interval; (**b**) distribution of credibility in the 1.1–1.2 m interval; (**c**) distribution of credibility in the 1.2–1.4 m interval.

The credibility achieved by fitting the different interval segments was plotted in Figure 9, and the standard deviation of each interval is calculated separately. The standard deviations of 1.0–1.1, 1.1–1.2, and 1.2–1.4 m were 0.32, 0.32, and 0.33, respectively. There was no significant difference in the standard deviation of the three intervals, which indicated that the fitting method was not affected by the selected interval.

## 4. Discussion

**Figure 10:**
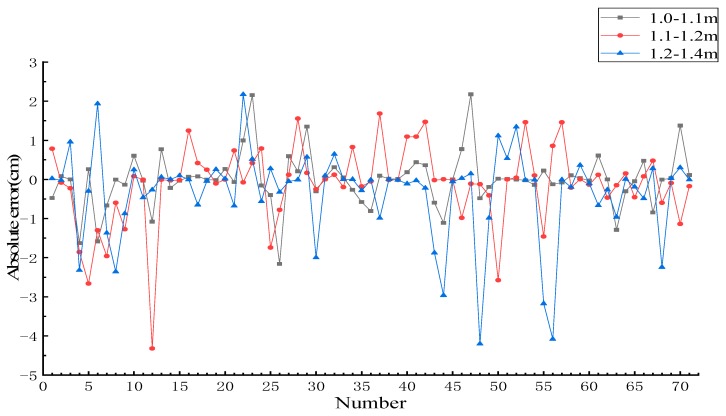
Distribution of absolute errors in different intervals.

For the statistical error distribution, the absolute errors of the fitting in each interval are plotted in Figure 10. It can be seen from Figure 10 that the absolute error fluctuation is the smallest in the 1.0–1.1 m interval, and the absolute error fluctuation is largest in the 1.2–1.4 m interval. The mean formula of the absolute error is as follows:

(1) $$Mean\;of\;absolute\;error=\frac{\sum_{n=1}^{71}\left|absolute\;error\right|}{71}$$

The variance and mean statistical results of the absolute error of each interval are shown in Table 1. It can be seen from the table that the variance and mean of the absolute error in the interval of 1.0–1.1 m are smaller than those in the other intervals, and the corresponding variance and mean are 0.50 and 0.43 cm, respectively.

**Figure 11:**
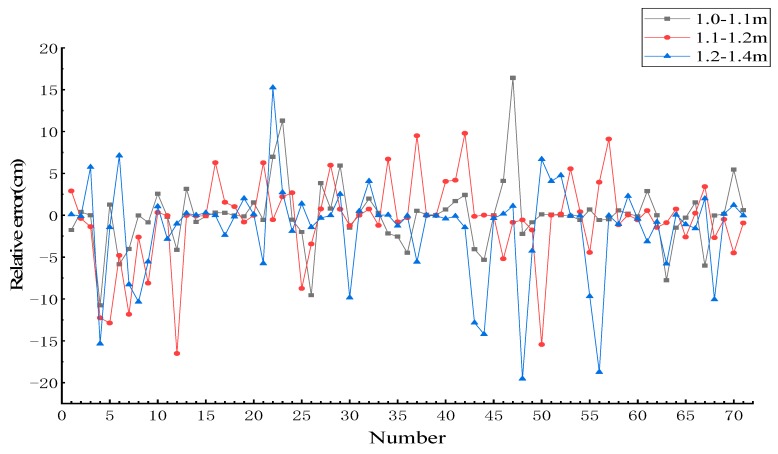
Distribution of relative errors in different intervals.

To further evaluate the resulting fitting error, the relative error of estimation DBH of each interval is plotted in Figure 11. The relative error fluctuation of the 1.0–1.1 m interval is the smallest, and the relative error fluctuation is the largest in the interval of 1.2–1.4 m. The formula for the mean relative error is as follows:

(2) $$Mean\;of\;relative\;error=\frac{\sum_{n=1}^{71}\left|relative\;error\right|}{71}$$

The statistical results for the variance and mean of the relative error of different intervals are shown in Table 2. The variance and mean of the interval of 1.0–1.1 m are the smallest, the corresponding variance is 15.09, and the mean is 2.27%. Combined with the analysis results regarding the absolute error, it can be concluded that in the experimental region, the error of the estimation DBH in the interval of 1.0-1.1 m is small, meaning that this can be used as the final experimental result.

Whether the error of the estimation DBH is related to the credibility was determined, and the correlation coefficient between the credibility and the absolute error was obtained. The results are shown in Table 3. The correlation coefficients of the confidence and absolute error for the 1.0–1.1, 1.1–1.2, and 1.2–1.4 m interval are 0.05, −0.03, and −0.01, respectively, and the correlation between the two physical quantities is not strong. It can also be concluded that the fitting method based on RANSAC was not affected by the number of points required for the fitting circle, indicating the robustness of the algorithm.

The RMSE values of the three different intervals were calculated. The REME values for 1.0–1.1, 1.1–1.2, and 1.2–1.4 m are 0.70, 1.00, and 1.18 cm, respectively. The RMSE of the estimation DBH in the interval of 1.0–1.1 m is the smallest.

According to the above error analysis, the trees were extracted from the three-dimensional point cloud map, and the interval of 1.0–1.1 m was selected as the interval where the DBH was located. The error of estimation DBH is the smallest.

**Figure 12:**
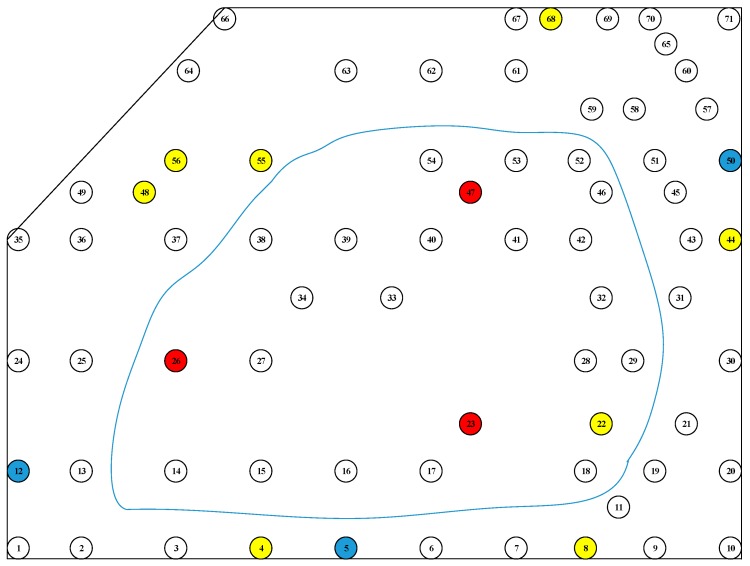
The absolute value of the absolute error of the trunk is greater than the position distribution of 2 cm. Red represents the interval of 1.0–1.1 m, blue represents the interval of 1.1–1.2 m, and yellow represents the interval of 1.2–1.4 m.

The absolute error is taken as an absolute value, and the trees with a value greater than 2 cm were identified in the schematic diagram, as shown in Figure 12. The corresponding intervals for the red, blue, and yellow marks are 1.0–1.1, 1.1–1.2, and 1.2–1.4 m, respectively. It can be seen that the trees corresponding to each interval error greater than 2 cm are different. The influencing factors can be attributed to the following factors: the speed of the walking process causing the equipment to shake, resulting in an inconsistent density of the instrument collected point cloud, affecting the fitting accuracy; the jitter caused by the fluctuation of the ground; and a DBH that is not in the selected interval. In the estimation results, the overall error was small, which meets the requirements of forest mapping.

Finally, a three-dimensional map of the forest area was successfully generated, and the DBH value of the trees was obtained. However, there was a lack of location for trees in the forest area. Thus, future work will mainly focus on the localization of trees via merging GPS information into the device, and the development of corresponding algorithms.

## 5. Conclusions

In this study, a designed handheld 3D scanning device was developed combining LiDAR, IMU, and other auxiliary equipment, based on which a three-dimensional map of a forest environment was generated. The DBH of the 71 trees in the forest area was fitted and extracted using RANSAC algorithm. The results demonstrated that the mean absolute and relative errors of DBH were 0.43 cm and 2.27%, with the corresponding variances of 0.50 and 15.09, respectively. The RMSE was 0.70 cm. The overall error satisfied the requirements of forestry mapping. GPS will be blended into the equipment in order to locate an individual tree, in order to expand the information of the map in future.

Three-dimensional map reconstruction technology based on SLAM is widely used in the forestry field. In the future, the equipment will be carried on a UAV for large-scale forest area surveying and mapping, and the scope of the application will be extended to agricultural and forestry contexts such as standardized orchards and vineyards.

## Figures and Tables

**Table 1:** Variance and mean of the absolute error of different intervals.

Interval	1.0–1.1 m	1.1–1.2 m	1.2–1.4 m
Variance	0.50	1.02	1.30
Mean of absolute error	0.43 cm	0.63 cm	0.68 cm

**Table 2:** Variance and mean of the relative error of different intervals.

Interval	1.0–1.1 m	1.1–1.2 m	1.2–1.4 m
Variance	15.09	25.31	32.26
Mean of relative error	2.27%	3.12%	3.45%

**Table 3:** Correlation coefficient between confidence and absolute error.

Interval	1.0–1.1 m	1.1–1.2 m	1.2–1.4 m
Correlation coefficient	0.05	−0.03	−0.01
